# Ischemic stroke in patients with POEMS syndrome: a case report and comprehensive analysis of literature

**DOI:** 10.18632/oncotarget.20131

**Published:** 2017-08-10

**Authors:** Fang-Wang Fu, Jie Rao, Yuan-Yuan Zheng, Hui-Lin Wang, Jian-Guang Yang, Guo-Qing Zheng

**Affiliations:** ^1^ Department of Neurology, The Second Affiliated Hospital and Yuying Children’s Hospital of Wenzhou Medical University, Wenzhou city, China; ^2^ Department of Neurology, The Fifth Affiliated Hospital of Wenzhou Medical University, Lishui Central Hospital, Lishui City, China

**Keywords:** POEMS syndrome, ischemic stroke, vasculopathy, vascular endothelial growth factor, vessel wall imaging

## Abstract

**Background:**

POEMS syndrome is a rare multi-systemic disease characterized by polyneuropathy, organomegaly, endocrinopathy, monoclonal gammopathy, and skin changes. Arterial or venous thrombosis is a less-common complication of POEMS syndrome. Ischemic stroke has also been reported sporadically. However, the association between POEMS syndrome and ischemic stroke has not been entirely understood.

**Methods:**

A case of ischemic stroke caused by cerebral vasculitis in a patient with POEMS syndrome was presented. Then a comprehensive review and analysis of the literature were performed.

**Results:**

A total of 28 patients were identified. The common clinical manifestations of POEMS syndrome were rather non-specific in patients with ischemic stroke compared with those of patients without ischemic stroke. Twenty patients were found with multiple ischemic lesions (71.5%). In the 25 patients who had undergone the evaluation of cerebral arteries, nineteen patients (76.0%) were found with cerebral vasculopathy. Twelve patients (48.0%) had more than one cerebral artery involved. Ischemic events were documented in 8 patients even when they were undergoing all the therapy for ischemic stroke. Ten (55.6%) of the 18 patients who had survival data died within two years after stroke events.

**Conclusion:**

Comprehensive analysis of literature revealed several trends in patients with ischemic stroke and POEMS syndrome including a low survival rate and a preponderance of cerebral vasculopathy and multiple cerebral arteries affected. Ischemic stroke may be a poor outcome predictor in patients with POEMS syndrome. Further researches focusing on a larger cohort may help in better characterizing and treating this rare complication of POEMS syndrome.

## INTRODUCTION

POEMS syndrome, also called as Crow-Fukase syndrome or Takatsuki syndrome, is a rare paraneoplastic syndrome characterized by polyneuropathy, organomegaly, endocrinopathy, mono-clonal gammopathy, and skin changes [1, 2]. It’s associated with an underlying clonal plasma cell dyscrasia [1, 2]. Other important manifestations include Castleman’s disease, sclerotic bone lesions, papilledema, thrombocytosis, polycythemia, extravascular volume overload, pulmonary hypertension, clubbing, weight loss, thrombotic diatheses and vitamin B12 deficiency [1, 3, 4]. Although the pathogenesis of POEMS syndrome is still unclear, the overproduction of vascular endothelial growth factor (VEGF), as one of the major diagnostic criteria of POEMS syndrome [1], may contribute to most of the above clinical manifestations and correlates with the disease activity and response to therapy [5, 6]. VEGF targets vessel endothelial cell by increasing vascular permeability and promoting angiogenesis [7-9], which may result in arterial or venous thrombosis [10, 11]. Recently, ischemic stroke is attracting increasing attention as a less-recognized manifestation of POEMS syndrome and its 5-years risk is reported to be 13.4% [12]. However, only a few patients have been reported to have ischemic stroke. The multi-vessel anomaly was documented in several studies [13-16], but the pathogenesis of cerebral vasculopathy is not clear.

In the current study, we presented a case of ischemic stroke and cerebral vasculitis diagnosed by the radiographic feature of vasculitis pathophysiology using magnetic resonance vessel-wall imaging technique in a female patient with POEMS syndrome. Subsequently, a comprehensive analysis of published literature was undertaken to study the clinical features and pathogenesis of ischemic stroke in POEMS syndrome.

## CASE REPORT

A 65-year-old female from China was initially admitted to our hospital with a 2-year history of numbness and mild weakness in the distal of low extremities. She experienced chest tightness, cough, fatigue and edema in low extremities for three years with a diagnosis made by a local hospital involving serous pericarditis, multiple lymphadenopathies, bronchiectasis and silent lacunar infarction. She was receiving aspirin, furosemide and spironolactone daily after discharge. She had no relevant family history and medical history of diabetes mellitus, hypertension, hypercholesteremia, alcohol consumption, cigarette smoking and drug abuse. Noteworthy features were found in the review of systems including a 12kg weight loss and pigmentation of skin in the neck, chest, back and limbs during the past two years. The tentative diagnosis of distal symmetrical sensorimotor polyneuropathies was made after nerve conduction studies and the neurological examination on admission. Meanwhile the initial treatment of aspirin, atorvastatin and oral mecobalamin started. On the third hospital day, acute respiratory infection occured, then empirical antibiotics treatment, including penicillin and levofloxacin, was initiated for this patient.

She developed sudden onset of dysarthria and right hemiplegia on the midnight of the seven days after hospitalization. Positive physical examination revealed marasmus, splenomegaly, clubbed-finger, pitting edema in both lower extremities, bilateral papilledema and hyperpigmentation in neck, chest and four limbs. Several nontender enlarged lymph nodes were palpable in the fossa axillary, inguinal area, neck region and left postauricular region. Neurological examination was notable for dysarthria, right hemiplegia with Grade III muscle strength, absent tendon reflexes, and signs of distal symmetrical sensorimotor polyneuropathies in extremities.

Routine laboratory tests, including blood routine, biochemistry test and coagulation function test, were performed and the results were listed in Table 1. It was noteworthy for moderate anemia (Red blood cell count 3.24×10^12^/L, hemoglobin 85 g/L), thrombocytosis (Platelet count 512×10^9^/L), hyperhomocysteinemia (21.3 umol/L), hypoalbuminemia (35 g/L), hyperuricemia (525 umol/L), hyperfibrinogenemia (5.96 g/L), and increased blood urea nitrogen concentration (12.24 mg/L). Hypersensitive C-reactive protein (Hs-CRP, 15.82 mg/L) was slightly increasing, but the erythrocyte sedimentation rate was normal. Serum tumor marks and autoimmune antibodies were all negative. Serum hormone test was noteworthy for hypothyroidism (Free triiodothyronine (FT3, 1.73 pg/ml), free thyroxine (FT4, 0.78 ng/dl), thyroid stimulating hormone (TSH, 8.3 mIU/L) with negative antibody (thyroglobulin antibody, thyroid peroxidase antibody and thyrotrophin receptor antibody), elevated adrenocorticotropic hormone (ACTH, 150.00 pg/ml) and decreased 24-hours urine free cortisol (81.2 nmol/24h). Glycosylated hemoglobin and oral glucose tolerance test were normal. The opening pressure of cerebrospinal fluid notably increased to 300 mmH_2_O. Cerebrospinal fluid examination demonstrated elevated protein level (1.95 g/L) and elevated IgG level (29.0 mg/dL) with normal cell content, normal concentration of chloride and glucose, and negative oligoclonal band.

**Table 1:** Summary of laboratory findings in the present case

Laboratory tests	Normal/negative	Abnormal finding
Routine blood test		
White blood cell count	Normal	
Red blood cell test		RBC 3.24×10^12^/L, range: 3.5-5.0×10^12^/L. Hemoglobin 85 g/L, range: 110-150 g/L. Hematocrit 0.27, range: 0.37-0.47. MCV 83 fl, range: 80-100 fl. MCH 26.1 pg, range: 27-34 pg. MCHC 314 g/L, range: 320-360 g/L.
Platelet count		Thrombocytosis ( 512X10^9^/L, range: 100-300×10^9^/L)
Reticulocytes		Mile increased (1.6%, range: 0.5-1.5 %)
Test for anemia		Mild increased erythropoietin (22.29 IU/L, range: 15-20 IU/L) Decreased serum transferrin (120 mg/dL, range: 200-340 mg/dl ) Decreased serum iron (6.20 μmol/L, range: 9-27 μmol/L ) Decreased total iron binding capacity (40.8 μmol/L, range: 50-77 μmol/L )
Hs-CRP		Elevated (15.82 mg/L, range: 0-3 mg/L)
Liver function tests		Hypoalbuminemia (35 g/L, range: 40-55 g/L) Decreased serum prealbumin (112 mg/L, range: 200-400 mg/L )
Renal function tests		Increased serum urea nitrogen ( 12.24 mmol/L, range: 2.8-8.2 mmol/L)
Serum uric acid		Increased (525 umol/L, range: 90-357 umol/L)
Plasma homocysteine level		Hyperhomocystinemia (21.3 umol/L, range: 5-15 umol/L)
Lipid profile	Normal	
Troponin T	Normal	
Serum folate level		Decreased (3.39 ng/ml, range: 3.7-19.8 ng/ml)
Serum Vitamin B12 level		Decreased (101 pg/ml, range: 180-914 pg/ml)
ESR	Normal	
Antiphospholipid antibody	Negative	
Serum tumor marks	Negative	
ANA and ENA antibodies	Negative	
ANCA antibodies	Negative	
Complement C3 and C4	Negative	
ASO and RF	Negative	
Coagulation test		Hyperfibrinogenemia (5.96 g/L, range: 2-4 g/L)
Serum cystatin C level	Normal	
Hepatitis panels	Negative	
HIV antibodies and TPPA	Negative	
Cerebrospinal fluid		Increased CSF pressure (300 mmH_2_O, range: 80-180 mmH_2_O) Increased protein (1.95 g/L,range: 0-0.4 g/L) and IgG (29 mg/dl, range: 0.4-5.8 mg/dL)
Oligoclonal band	Negative	
Thyriod function		Decreased free T3 (1.73 pg/ml, range: 2.30-4.20 pg/ml) Decreased free T4 (0.78 ng/dl, range: 0.90-1.80 ng/dl) Increased TSH (8.3 mIU/L, range: 0.55-4.78 mIU/L)
Anti-thyroid antibodies	Negative	
Endocrinopathy panels		Increased ACTH (150.00 pg/ml, range: 7.2-63.3 pg/ml ) Decreased 24-hours urinary free cortisol (81.2 nmol/24h, range: 129.7-303.6 nmol/L)
TORCH panels	Negative	
Serum immunofixation		IgG kappa monoclonal protein
Urine immunofixation		Tiny amount of IgG kappa monoclonal protein
Serum VEGF		Significantly increased (7712 pg/ml, range:100-720 pg/ml)

MCV: mean corpuscular volume. MCH: mean corpuscular hemoglobin.

MCHC: mean corpuscular hemoglobin contentration.

Serum tumor marks: CEA, AFP, CA125, CA199, CYRFA21-1, SCC, NSE.

ANA: antinuclear antibodies. ENA: extractable nuclear antigen antibodies.

RF: rheumatoid factor. TPPA: treponema pallidum particle agglutination assay.

TSH: thyroid stimulating hormone. ACTH: adrenocorticotropic hormone.

TORCH: Toxoplasmosis, other diseases(including HIV, syphilis, and measles),rubella, cytomegalovirus and herpes simplex.

VEGF: vascular endothelial growth factor.

**Figure 1:**
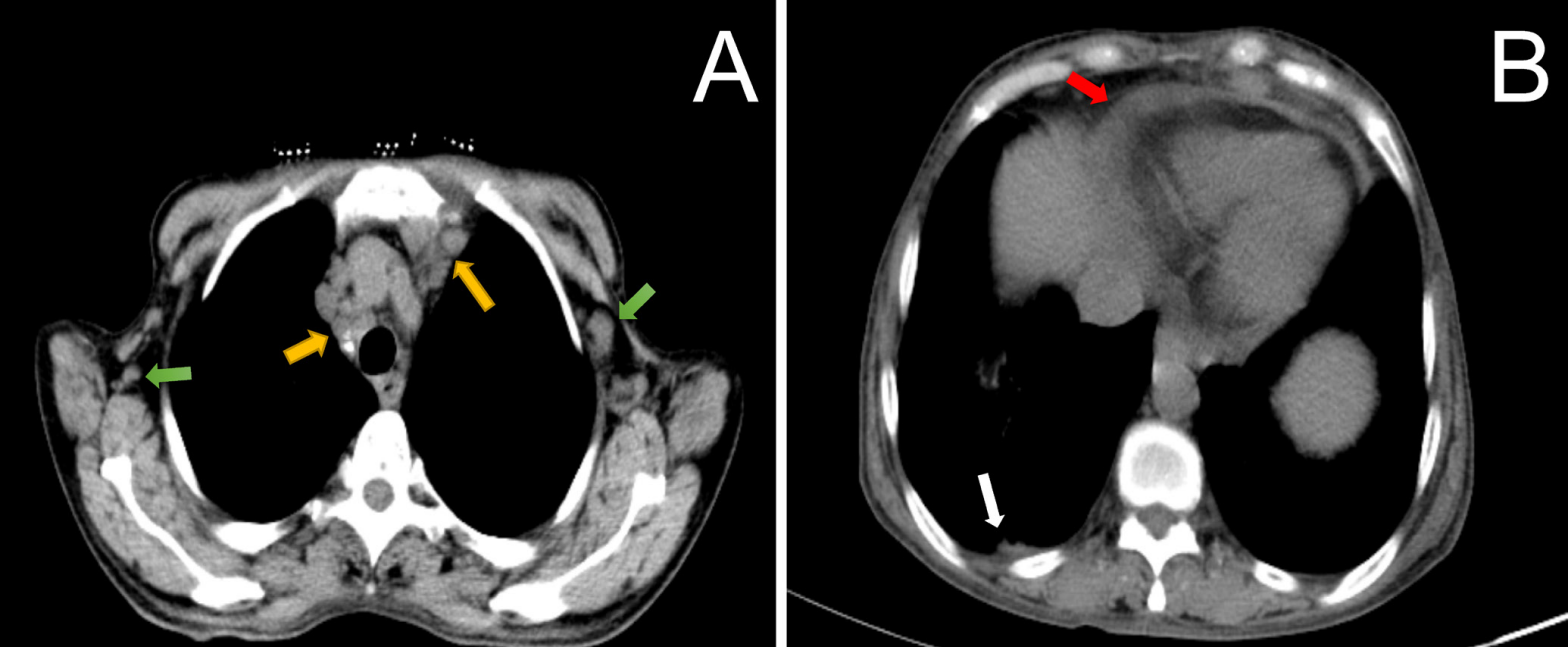
CT scan of the thorax showed multiple axillary (Figure 1A, green arrow), and mediastinal lymphadenopathy (Figure 1A, yellow arrow) in combination with right pleural effusion (Figure 1B, white arrow) and pericardial effusion (Figure 1B, red arrow)

**Figure 2:**
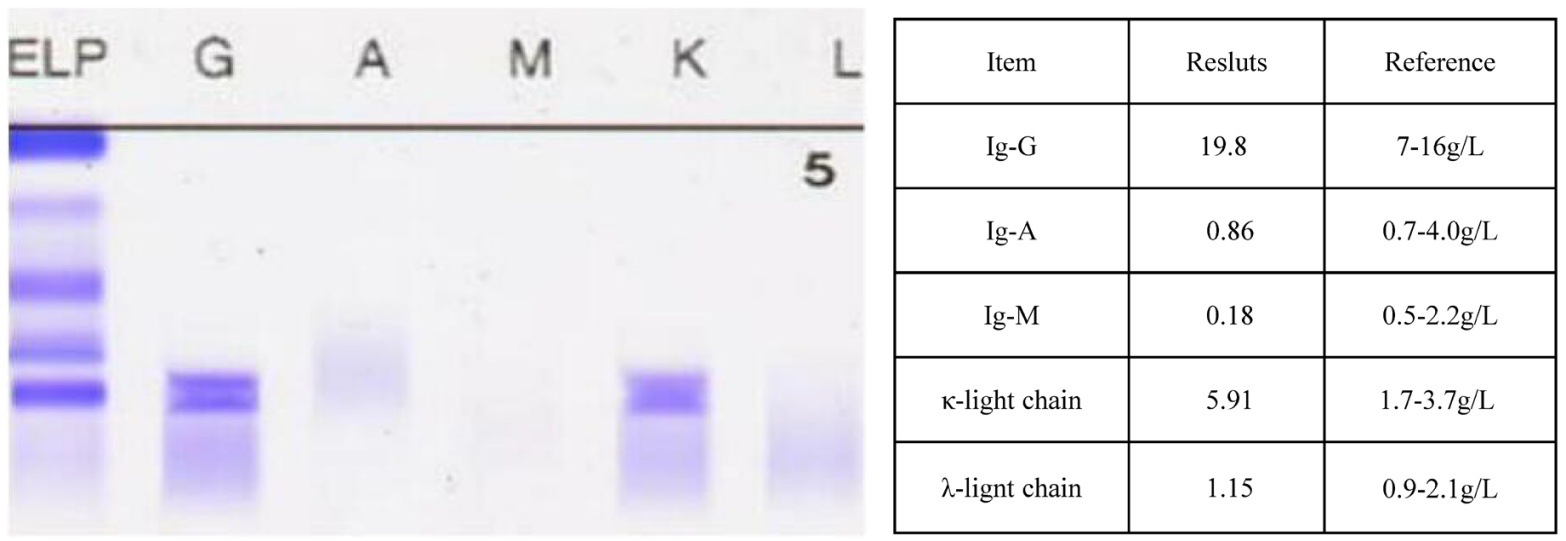
Serum immunofixation electrophoresis revealed an IgG kappa monoclonal protein(Figure 2A) with increased IgG and free kappa chain (Figure 2B)

The nerve conduction study and electromyogram revealed diffuse distal sensorimotor polyneuropathies including both axonal and demyelinating neuropathies. Splenomegaly, lymphadenopathy, moderate ascites were confirmed by sonography. CT scan showed multiple mediastinal lymphadenectasis, right pleural effusion and bronchiectasis in the right middle lobe and left upper lobe (Figure 1). No evidence of sclerotic bone lesions was found in whole bone scan. Serum vascular endothelial growth factor was dramatically increasing (7712 pg/mL). To detect monoclonal gammopathy, serum and urine immunofixation electrophoresis were performed. It was noteworthy in serum for an IgG kappa monoclonal protein with increased IgG and free kappa chain (Figure 2). The presence of a very tiny amount of IgG kappa monoclonal protein was detected in urine immunofixation.

**Figure 3:**
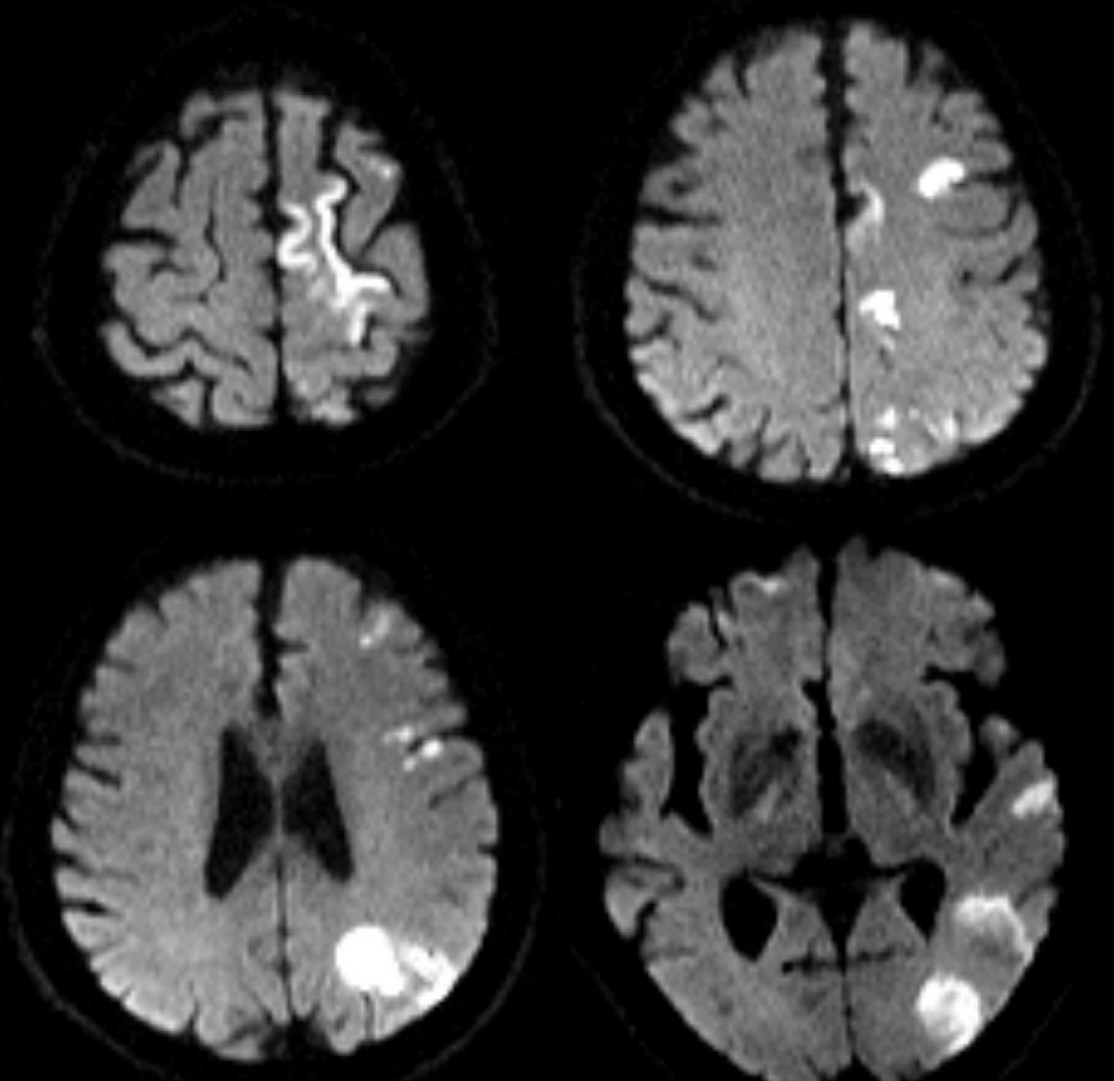
Axial DWI sequences showed multiple acute infarction lesions in end artery border-zone of left cerebral hemisphere

**Figure 4:**
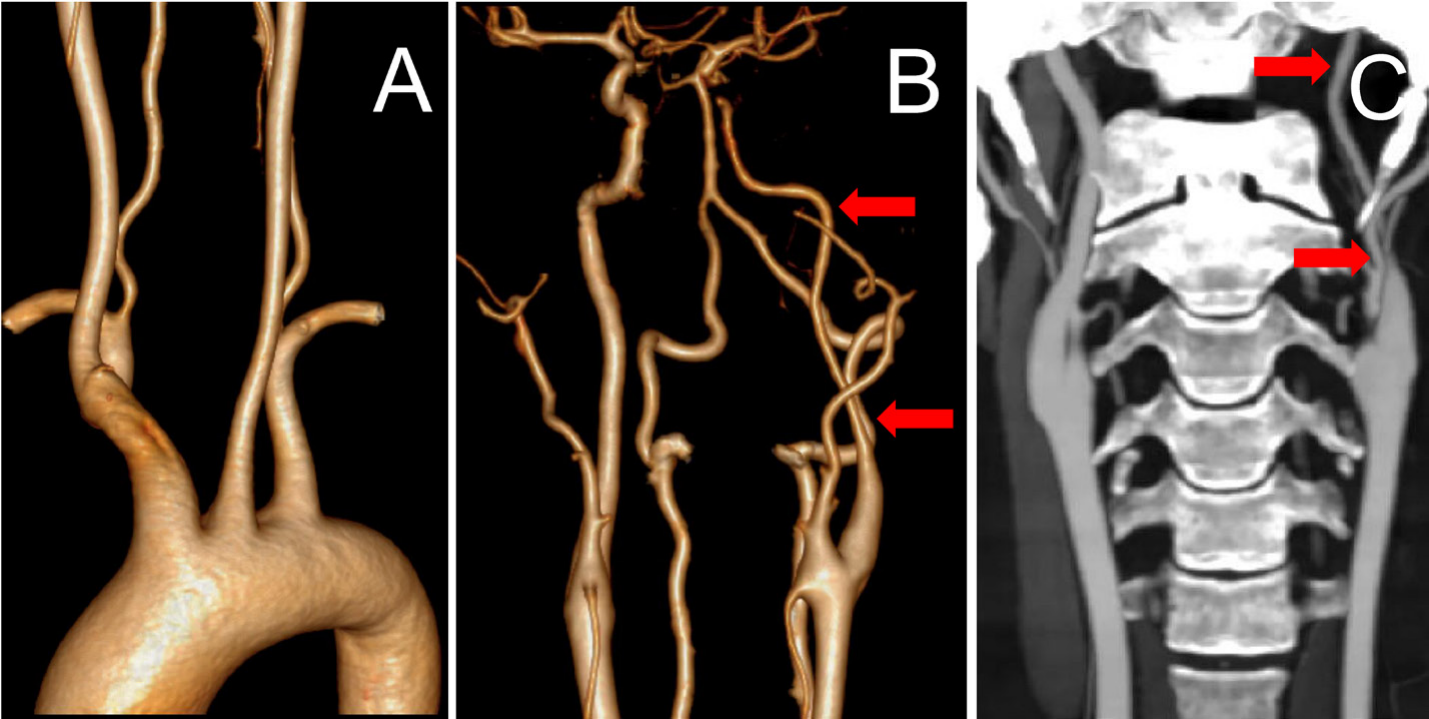
CT angiography showed a great aortic arch and normal artery (Figure 4A) except diffuse and moderate stenosis in the large vessels from the origin of left ICA to the M1 segment of the left MCA (Figure 4B and 4C, red arrow)

**Figure 5:**
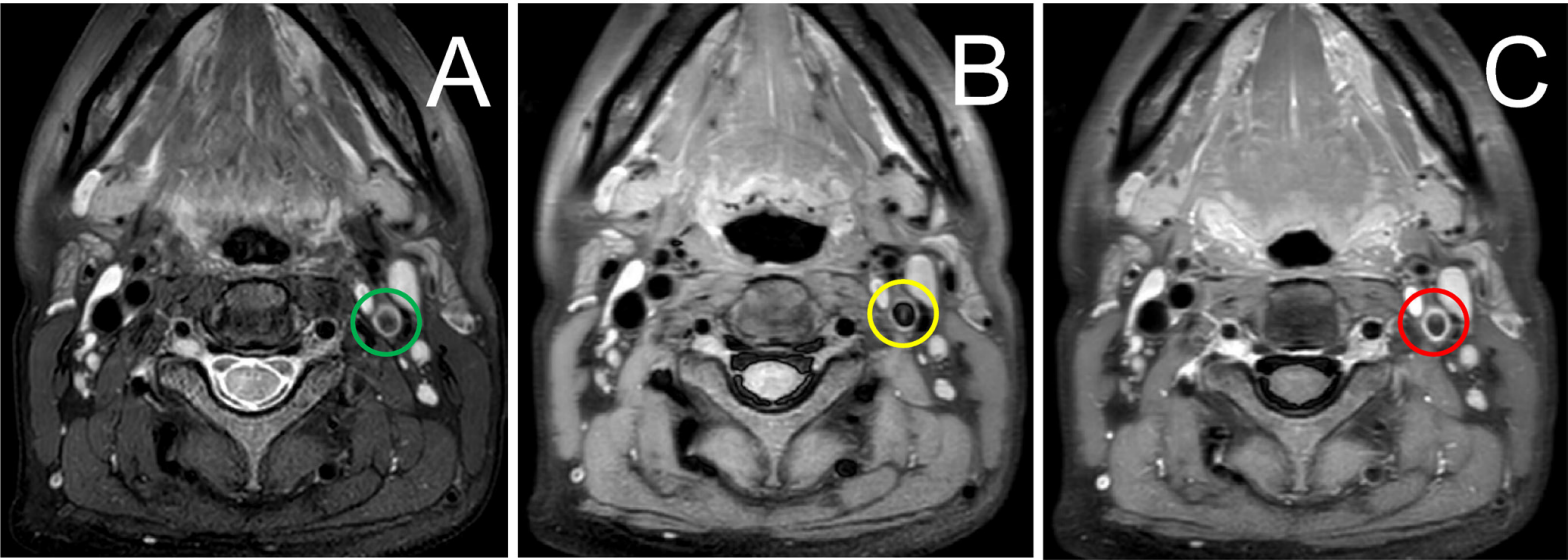
High-resolution T2-weighted imaging showed concentric hyperintensity and thickening in the vessel wall of left ICA and (Figure 5A, green circle) High-resolution, black-blood MRI before (Figure 5B, yellow circle) and after (Figure 6C, red circle) gadolinium injection demonstrating a strong, smooth, and concentric vessel wall enhancement in left ICA.

The brain magnetic resonance imaging (MRI) was done three days after stroke onset. The diffusion-weighted imaging showed multiple acute infarction lesions in end artery border-zone of the left cerebral hemisphere (Figure 3). Transthoracic echocardiography revealed a moderate pulmonary arterial hypertension with a systolic pressure of 53 mmHg and mild pericardial effusion of 4 mm thickness without the presence of abnormal valvular structure and decreased left ventricular ejection fraction. There was no evidence for foramen ovale, atrial myxoma, left atrial appendage thrombus and any aortic pathologies in transesophageal echocardiography. Transcranial doppler, Holter monitoring, and carotid artery ultrasound failed to detect the embolic source. CT angiography showed a great aortic arch and normal artery except diffuse and moderate stenosis in the large vessels from the origin of left internal carotid artery (ICA) to the M1 segment of the left middle cerebral artery (MCA) (Figure 4). Strong enhancement of thickened vessel wall in left ICA was observed with high-resolution black-blood gadolinium-enhanced T1-weighted images, suggestive of vasculitis (Figure 5).

**Figure 6:**
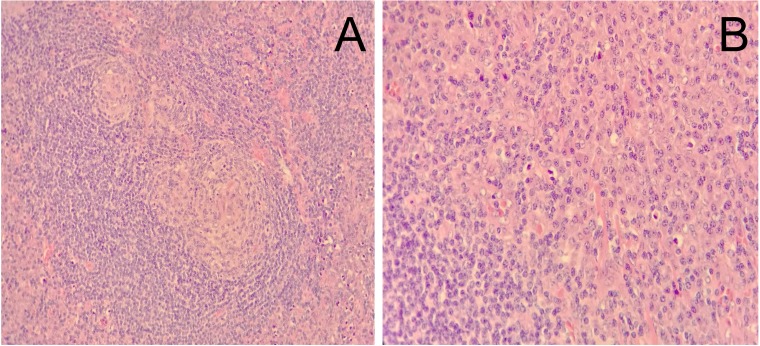
Histological examination of cervical lymph node revealed infiltration of plasma cells in the interfollicular area (original magnification A: ×200, B: ×400)

Multicentric plasma cell variant of Castleman’s disease was diagnosed with biopsy of a cervical lymph node (Figure 6). Bone marrow biopsy in the back of the pelvic demonstrated slightly increased plasma cell (about 4% of marrow elements) with a small account of atypical cells which were positive for CD3, CD38, CD138, CD79a, CD43, Ki-67 and kappa light chain and negative for CD20 and CD56. But it failed to provide adequate evidence of a definitized myeloma.

We diagnosed her with POEMS syndrome, Castleman’s disease and ischemic stroke due to cerebral vasculitis. Since single antiplatelet therapy showed no effect on stopping ischemic recurrence in many previous reports, we changed the initial single antiplatelet therapy into dual antiplatelet therapy with aspirin (100 mg/day) and clopidogrel (75 mg/day) for 14 days, and then switched to aspirin 100 mg/day. After refusing autologous stem cell transplantation, the treatment with mecobalamin (1000 ug/day), dexamethasone (20 mg/day on day 1, 8, 15, and 22 of each 28-day cycle) and thalidomide (100 mg/day in first week, 150 mg/day in second week, then 200 mg/day) was started. The symptoms of dysarthria and right hemiparesis relieved after a week without any side effects of drugs. At a 6-month follow-up, we observed clinical improvement of numbness, weakness, edema and no recurrence of stroke with a reduced VEGF level (1324 pg/L).

## LITERATURE SEARCH

To retrieve all relevant studies on correlation between POEMS syndrome and ischemic stroke, we conducted a comprehensive search of Pubmed, Web of Science, EMBASE, China National Knowledge Infrastructure (CNKI), Wanfang Database and China Scientific Journal Database (VIP) using the terms “POEMS syndrome” (OR “Crow-Fukase syndrome”, “Takatsuki syndrome”) and “ischemic stroke” (OR “cerebral infarction”, “brain infarction”, “brain ischemia”, “transient ischemic attack”). The studies including case report and case series published from database inception to February 2017 were included without any language restriction. In addition, articles which didn’t have a decomposition of case series into a pattern in which cases could be analyzed individually were excluded, because we should obtain the clinical data such as age, gender, age of stroke onset, initial symptoms, time intervals between stroke onset and POEMS syndrome onset, the location of cerebral infarction, offending vessel, laboratory tests, MRI or CT image, angiography, biopsy, treatment and outcome data from each case. “Symptom improved” was used as good prognosis measure because it was the most common terms used by authors to describe the patient’s outcome, while “died” or “deterioration” was used as poor prognosis measure. Every literature was reviewed by two researchers independently.

## RESULTS

To the best of our knowledge, 24 reports, including three small series and 21 single reports describing 28 cases of POEMS syndrome complicated with ischemic stroke, were identified after exclusion of the reports with incomplete data or duplicate data. All the available data were summarized in Table 2a-2d by reviewing the full texts of eligible reports. The comparison of clinical feature in the present study and two previous series was showed in Table 3.

**Table 2a:** Basic characteristics in patients with POEMS syndrome and ischemic stroke

	Lesprit et al. 1996 [14]	Hori et al. 1999 [38]	Zhang et al. 2002 [39]	Kang et al. 2003 [21]			Erro et al. 2003 [20]
Age (years)	53	51	44	42	48	52	65
Sex	Female	Female	Male	Male	Female	Female	Female
Initial symptoms	Organomegaly	Lymphadenopathy	Skin changes	N.A.	N.A.	Polyneuropathy	N.A.
POEMS to Stroke time	24 months	11 years	12 months	N.A.	N.A.	4 months	29 months
Symptoms of stroke	Monocular blindness	Aphasia and right hemiplegia	Left hemiplegia	Dysarthria Right arm monoparesis	Weakness of all limbs	Slurred speech	Right monocular blindness
Infraction territory	Right ICA territory	Left MCA territory	Right MCA territory	left MCA end BZI Left MCA/PCA BZI	Bilateral end artery BZI Bilateral PCA territory	Left MCA/PCA BZI	Right ICA territory
Cerebral vasculopathy	Right ICA occlusion Left ICA stenosis	Bilateral ICA occlusion	Right MCA occlusion Diffuse vessels stenosis	Inceased MFV of left ACA, MCA and right ICA	Left MCA stenosis	Left ICA stenosis	Right ICA occlusion
Vessels evaluation methods	DSA	DSA	Ultrasonography	TCD	MRA	MRA	MRA
Extracerebral vasculopathy	SMA and left SA stenosis	N.A.	-	Coronary artery	Lower extremity artery	N.A.	Pulmonary artery
Events (TIA, Stroke)	1	1	1	1	1	1	2
Risk factors	-	Hypertension	-	Smoking	-	Hypertension	-
Relevant comorbidities	-	-	-	Myocardial infarction	Left foot gangrene	-	Pulmonary embolism
Medications before stroke	-	Cyclophosphamide and prednisone	Dexamethasone	Aspirin	N.A.	-	Oral anticoagulation IVIg and prednisone
Polyneuropathy	+	+	+	+	+	+	+
Monoclonal protein	IgA-λ	IgG-λ	IgA-λ	-	+	+	IgG-λ
Castleman disease	Not performed	+	Not performed	Not performed	+	Not performed	Not performed
Scleroticbone lesions	+	-	+	-	-	-	-
Organomegaly	Hepatosplenomegaly Lymphadenopathy	Hepatosplenomegaly Lymphadenopathy	Hepatomegaly	Hepatosplenomegaly Lymphadenopathy	Hepatomegaly Lymphadenopathy	-	Splenomegaly
Extravascular volume overload	Pleural effussion Ascites Peripheral edema	Peripheral edema	Peripheral edema	-	Ascites	Pericardial effusion	-
Endocrinopathy	Glucose intolerance	Hypothyroidism Amenorrhoea	Gynaecomastia Hyperprolactinemia	Hypothyroidism	Hypothyroidism	Hypothyroidism	Hypothyroidism
Skin changes	+	+	+	+	+	+	+
Papilledema	-	-	-	-	-	-	-
Thrombocytosis	1000×10^9^/L	-	612×10^9^/L	695×10^9^/L	-	-	560×10^9^/L
Hyperfibrinogenemia	-	-	-	6.03g/L	5.94g/L	3.61g/L	-
Antibodies	-	-	-	-	-	-	-
Other abnormalities	Elevated IL-1B	Mild anaemia Elevated CSF protein Elevated CSF pressure Elevated ESR Decreased Ccr	Polycythemia Elevated CSF protein Weight loss (15kg in 12 months)	Elevated CRP	Elevated CRP	Elevated CRP Pulmonary hypertension	Elevated CSF protein Pulmonary hypertension
Stroke treatment	Aspirin and bypass graft	Aspirin	Aspirin	N.A.	N.A.	N.A.	Oral anticoagulation and aspirin
POMES treatment	Cyclophosphamide	Melphalan and prednisolone	Dexamethasone	N.A.	N.A.	N.A.	Azathioprine and prednisone
Response to treatment	Deteriorated	Improved	Deteriorated	N.A.	N.A.	N.A.	N.A.
Survival time	Died after 2 years	N.A.	Died 12 months later	N.A.	N.A.	N.A.	N.A.

POEMS to Stroke time: Time from POEMS syndrome onset to stroke onset.

ICA: internal carotid artery. MCA: middle cerebral artery. ACA: anterior cerebral artery. PCA: posterior cerebral artery. BZI: border-zone infarction.

SA: subclavian artery. SMA: superior mesenteric artery.

DSA: digital subtraction angiography. MRA: magnetic resonance angiography. CTA: computed tomography angiography. TCD: transcranial doppler. MFV: mean flow velocity.

Ccr: endogenous creatinine clearance rate. IVIg: Intravenous immune globulin.

N.A.: not available.

**Table 2b:** Basic characteristics in patients with POEMS syndrome and ischemic stroke

	Rolsser et al. 2004 [22]	Wang et al. 2006 [30]	Garcia et al. 2007 [13]		Huang et al. 2007 [23]	Lee et al. 2007 [14]	Du et al. 2008 [31]
Age (years)	32	61	49	50	42	41	65
Sex	Female	Male	Male	Female	Female	Male	Female
Initial symptoms	Edema in limbs	Polyserositis	Polyneuropathy	N.A	Diarrhea	Polyneuropathy	Skin changes
POEMS to Stroke time	6 months	12 months	24 months	12 months	5 months	16 months	24 months
Symptoms of stroke	Right hemiplegia	Left hemiplegia	Aphasia Right hemianesthesia	Left hemianesthesia	Left hemiplegia Coma	Dysarthria Left hemiparesis	Right hemiplegia
Infraction territory	Left basal ganglia	Right MCA BZI	Left MCA end artery	Multiple bilateral infractions	Rihgt ICA and Left ACA	Right MCA territory	Left MCA BZI
Cerebral vasculopathy	Left MCA occlusion Left ACA stenosis	Normal	Left MCA occlusion Right MCA and ACA stenosis	Multiple bilateral arteries stenosis	N.A.	Bilateral MCA stenosis Right ICA stenosis	Normal
Vessels evaluation methods	DSA	Ultrasonography	DSA	DSA	N.A.	MRA	Ultrasonography
Extracerebral vasculopathy	N.A.	-	N.A.	Vegetation or thrombus in aortic outflow region	N.A.	SMA stenosis Left renal artery stenosis	-
Events (TIA, Stroke)	10 TIAs, 2 infractions	1	2	at least 3	1	1	1
Risk factors	Hypertension	-	Hypertension Hyperlipidemia	Hypertension Diabetes, smoking	-	-	-
Relevant comorbidities	-	-	Ischemic stroke	-	-	-	-
Medications before stroke	Antihypertensive Levothyrocine	Diuretic	Aspirin	Aspirin, prednisone and cyclophosphamide	Antithyroid drugs	Prednisolone	Diuretic
Polyneuropathy	+	+	+	+	+	+	+
Monoclonal protein	IgA-λ	IgG-λ	IgA-λ	IgG-λ	IgA-κ	IgA-λ	λ-light chain
Castleman disease	+	Not performed	+	+	+	-	Not performed
Scleroticbone lesions	-	+	+	-	-	+	+
Organomegaly	Hepatosplenomegaly Lymphadenopathy	Splenomegaly Lymphadenopathy	Splenomegaly Lymphadenopathy	Lymphadenopathy	Hepatosplenomegaly Lymphadenopathy	Hepatosplenomegaly Lymphadenopathy	Splenomegaly Lymphadenopathy
Extravascular volume overload	Pericardial effusion Pleural effusion Peripheral edema	Pericardial effusion Pleural effusion Ascites Peripheral edema	Pericardial effusion Pleural effusion Ascites	Peripheral edema	Pericardial effusion Ascites Peripheral edema	Ascites Peripheral edema Pericardial effusion	Pericardial effusion Pleural effusion Ascites Peripheral edema
Endocrinopathy	Hypothyroidism	Gynaecomastia Hyperprolactinemia	Hypogonadism Hypothyroidism	-	Hyperthyroidism	Hypogonadism Hypothyroidism	-
Skin changes	+	+	+	+	+	+	+
Papilledema	-	+	+	-	-	+	+
Thrombocytosis	-	374×10^9^/L	-	-	-	511×10^9^/L	-
Hyperfibrinogenemia	-	-	-	-	4.12g/L	-	-
Antibodies	-	-	ANA 1:160 peckled pattern	ANA	-	-	-
Other abnormalities	Hyperhomocystinemia Elevated CSF protein	Elevated CSF Protein Positive CSF oligoclonal bands	Elevated CRP and ESR Elevated CSF Protein Vitamin B12 deficiency Elevated VEGF and IL-6 Weight loss	Pancytopenia Hypoalbuminemia Elevated triglycerides Elevated ESR, D-dimer	Hypoalbuminemia Hyperglobulinemia Weight loss	Elevated CRP and ESR Elevated serum creatine	Positive CSF oligoclonal bands
Stroke treatment	First heparin Then clopidogrel and phenprocoumon	Aspirin	Aspirin	Aspirin then clopidogrel	N.A.	Anticoagulants (not specified)	Defibrase,then ozagrel, then aspirin
POMES treatment	First prednisone and chlorambucil, then VAD, then cyclophosphamide	Prednisone	First corticosteroids and rituximab, then dexamethasone and cyclophosphamide	First Prednisone and cyclophosphamide, then rituximab and methylprednisolone	Dexamethasone	Prednisone and melphalan	Prednisone
Response to treatment	Improved after cyclophosphamide	Deteriorated	Improved	Deteriorated	Improved	Deteriorated	Deteriorated
Survival time	At least 2 years	Died 6 months later	N.A.	N.A. “gradual deterioration”	N.A.	Died 4 weeks later	Died 6 months later

POEMS to Stroke time: Time from POEMS onset to stroke onset.

ICA: internal carotid artery. MCA: middle cerebral artery. ACA: anterior cerebral artery. PCA: posterior cerebral artery. SMA: superior mesenteric artery.

BZI: border-zone infarction. DSA: digital subtraction angiography. MRA: magnetic resonance angiography. TCD: transcranial doppler.

ANA: antinuclear antibody.

VAD: vincristin + doxorubicin + dexamethasone. IVIg: Intravenous immune globulin.

N.A.: not available.

**Table 2c:** Basic characteristics in patients with POEMS syndrome and ischemic stroke

	Zheng et al. 2008 [32]	Shi et al. 2010 [33]	Jing et al. 2011 [34]	Sommer et al. 2012 [24]	Dacci et al. 2013 [25]		Akyol et al. 2014 [15]
Age (years)	52	58	40	54	49	46	47
Sex	Male	Male	Male	Female	Male	Male	Female
Initial symptoms	Impotence	Polyserositis	Skin changes	Polyneuropathy	Ischemic stroke	Ischemic stroke	Polyneuropathy
POEMS to Stroke time	3 years	60 months	More than 2 years	2 months	12 months preceded POEMS onset	1 month preceded POEMS onset	2.5 months
Symptoms of stroke	Left upper limb palsy	Vertigo Left hemiplegia	Right hemiplegia	Right hemiparesis Global aphasia	Aphasia Right hemiparesis	Left facio-brachial palsy	Dysarthria Right-sided hemiparesis
Infraction territory	Right MCA territory	Right MCA territory	Multiple bilateral infractions	Left MCA and left MCA-ACA BZI	Left MCA BZI	Right PCA BZI	Bilateral parietal lobes
Cerebral vasculopathy	Bilateral carotid atherosclerosis	Normal	Left MCA occlusion	–	–	N.A.	Left ICA stenosisBilateral MCA occlusion
Vessels evaluation methods	MRA	Ultrasonography	DSA	Ultrasonography	Ultrasonography	N.A.	MRA
Extracerebral vasculopathy	–	–	–	Coronary artery	N.A.	N.A.	Pulmonary artery
Events (TIA,Stroke)	1	1	10	2	1	2	4-5 times per day
Risk factors	–	–	–	Hypertension, diabetes	Smoking	–	Hypertension, diabetes,hyperlipidemia
Relevant comorbidities	–	–	–	Myocardial infarction	PFO (underwent closure)	–	Hashimoto thyroiditis Pulmonary embolism
Medications before stroke	Prednisolone	Melphalan and prednisolone	Aspirin	Aspirin	N.A.	N.A.	Warfarin, aspirin, atorvastatin, gliclazide
Polyneuropathy	–	–	–	–	–	–	–
Monoclonal protein	–	IgA-λ	IgG-κ	IgG-λ	IgA-λ	IgG-λ	–
Castleman disease	Not performed	Not performed	–	Not performed	Not performed	Not performed	Not performed
Scleroticbone lesions	–	–	–	–	–	–	–
Organomegaly	Hepatosplenomegaly	Splenomegaly	Lymphadenopathy	Hepatosplenomegaly	Hepatosplenomegaly	Splenomegaly	Hepatosplenomegaly
Extravascular volume overload	Pleural effusionPeripheral edema	Pericardial effusion Pleural effusion Peripheral edema	Pleural effusion Peripheral edema	Pleural effusion	–	Pleural effusion	Pericardial effusion Peripheral edema
Endocrinopathy	Hypothyroidism Gynaecomastia Low testosterone Hyperprolactinemia	Hyperprolactinemia Low testosterone Hypercortisolism	Impotence	Hypothyroidism	–	–	Subclinical hypothyroidism
Skin changes	–	–	–	–	–	–	–
Papilledema	–	–	–	–	–	–	–
Thrombocytosis	386×10^9^/L	–	–	–	511x×10^9^/L	483×10^9^/L	687×10^9^/L
Hyperfibrinogenemia	–	–	6.32g/L	–	–	–	4.18g/L
Antibodies	–	–	–	–	–	–	–
Other abnormalities	Elevated CSF protein Elevated CSF pressure Glucose intolerance	Hyperglobulinemia Hypoalbuminemia Microalbuminuria	Polycythemia Hyperglobulinemia Albuminuria	Weight loss (10kg)	Hyperhomocystinemia Elevated CSF Protein	Elevated serum VEGF Elevated CSF protein Elevated CRP andESR	Orthostatic hypotension Elevated CSF protein Pulmonary hypertension Severe tricuspid regurgitation
Stroke treatment	Aspirin	N.A.	Aspirin	Aspirin and clopidogrel	Aspirin	Aspirin	Warfarin, aspirin, atorvastatin
POMES treatment	First prednisone, then prednisone and azathioprine	Melphalan and prednisone	Corticosteroids	Prednisone and radiotherapy	Prednisone and IVIg,then ASCT	First PE and IVIg,then Rev/Dex, then ASCT	Melphalan and prednisone, then cyclophosphamide
Response to treatment	Deteriorated	Improved	Improved	Deteriorated	Improved	Improved	Deteriorated
Survival time	Died 1 year later	N.A.	N.A.	Die 1 day later	at least 12 months	at least 6 months	Sudden cardiac death after two cycles treatment

POEMS to Stroke time: Time from POEMS onset to stroke onset.

ICA: internal carotid artery. MCA: middle cerebral artery. ACA: anterior cerebral artery. PCA: posterior cerebral artery. BZI: border-zone infarction.

DSA: digital subtraction angiography. MRA: magnetic resonance angiography.

IVIg: Intravenous immune globulin. ASCT: autologous stem cell transplant. PE:plasma-exchange. Rev/Dex: lenalidomide and dexamethasone.

N.A.: not available.

**Table 2d:** Basic characteristics in patients with POEMS syndrome and ischemic stroke

	Yu et al. 2015 [26]	Sun et al. 2015 [36]	Han et al. 2016 [27]	Rajan et al. 2016 [16]	Yagihi et al. 2016 [28]	Liu et al. 2016 [37]	Wang et al. 2016 [29]
Age	24	46	33	36	32	37	68
Sex	Male	Female	Male	Female	Male	Male	Male
Initial symptoms	Fever and polyserositis	Polyneuropathy	Skin changes and impotence	Polyneuropathy	Headaches	Polyneuropathy	Ischemic stroke
POEMS to Stroke time	2 months	9 months	12 months	3 months	11 months preceded POEMS onset	35 months	1 month preceded POEMS onset
Symptoms of stroke	Numbness of the upper extremities Blurred vision	Acute cognitive impairment	Intermittent amaurosis of left eye	Aphasia Left facial palsy	Right monocular blindness	Left monocular blindness Right side hemiparesis	Right hemiparesis
Infraction territory	First left ventricle,then right ventricle	Bilateral end artery BZI	Left ICA territory	Left MCA end artery and BZI	RightMCA internal BZI Ophthalmic artery	Left MCA BZI and ophthalmic artery	Left MCA-ACA BZI
Cerebral vasculopathy	N.A.	Right ICA occlusion Left ACA stenosis Right ICA stenosis	Left ICA occlussion	Bilateral ICA stenosis Left PCA occlusion	Right ICA occlusion	Left ICA stenosis	Normal
Vessels evaluation methods	N.A.	MRA	CTA	MRA	DSA	MRA	CTA
Extracerebral vasculopathy	–	–	–	–	Splenic artery	–	–
Events (TIA, Stroke)	At least 4 times	1	1	1	More than 4 times	1	3
Risk factors	–	–	–	–	Smoking	–	–
Relevant comorbidities	–	–	–	–	–	–	–
Medications before stroke	Antitubercular agents	Corticosteroids	–	–	–	Radiotherapy	Aspirin and atorvastatin
Polyneuropathy	–	–	–	–	–	–	–
Monoclonal protein	–	IgG-λ	λ-light chain	IgA-λ	IgA-λ	IgG-λ	IgA-λ
Castleman disease	–	Not performed	–	Not performed	–	Not performed	Not performed
Scleroticbone lesions	–	–	–	–	–	–	–
Organomegaly	Lymphadenopathy	Splenomegaly Lymphadenopathy	Splenomegaly Lymphadenopathy	Hepatosplenomegaly	Hepatosplenomegaly Lymphadenopathy	–	Splenomegaly Lymphadenopathy
Extravascular volume overload	Pericardial effusion Pleural effusion Peripheral edema	Pleural effussion	–	Ascites Peripheral edema	Ascites Peripheral edema Pericardial effusion	Pericardial effusion Peripheral edema	Peripheral edema
Endocrinopathy	Hypothyroidism Gynecomastia Hypotestosterone Hyperprolactinemia	Hypothyroidism	Hypothyroidism Hyperprolactinemia	–	Hypothyroidism Hypogonadism Low testosterone	Gynaecomastia Impotence Hyperprolactinemia	Hypothyroidism Low testosterone Hyperprolactinemia
Skin changes	–	+	–	–	–	+	–
Papilledema	–	–	–	–	–	–	–
Thrombocytosis	565×10^9^/L	427×10^9^/L	329×10^9^/L	697×10^9^/L	700×10^9^/L	379×10^9^/L	452×10^9^/L
Hyperfibrinogenemia	–	–	–	–	–	–	–
Antibodies	–	–	–	–	–	–	–
Other abnormalities	Elevated serum creatine Elevated CSF protein Elevated CSF pressure Weight loss (15kg in 60 days)	Elevated CRP and ESR Elevated serum VEGF Elevated CSF Protein Weight loss (3kg in 1 month)	Polycythemia Hyperuricemia Dyslipidemia Hyperphosphatemia Microalbuminuria	Polycythemia Elevated CSF protein	Elevated CRP and ESR Elevated serum VEGF Elevated CSF Protein Proteinuria Weight loss	PolycythemiaLeukocytosis Elevated CRP Elevated CSF Protein	Elevated serum VEGF Elevated ESR Weight loss (3kg in 2 months)
Stroke treatment	N.A.	Aspirin	Low molecular heparin	Antiplatelet drug	Aspirin and verapamil	Aspirin	Aspirin, clopidogrel atorvastatin
POMES treatment	Lenalidomideand dexamethasone	Lenalidomide and dexamethasone	Dexamethasone and melphalan	Thalidomide and dexamethasone	Methylprednisolone,then prednisone,then prednisone and cyclophosphamide	First melphalan-based chemotherapy,then ASCT	Melphalan and prednisone
Response to treatment	Deteriorated	Improved	N.A.	N.A.	Deteriorated	Improved	Improved
Survival time	Died two weeks later	At least 12 months	At least 6 months	N.A.	Died 2 years later	At least 12 months	At least 24 months

POEMS to Stroke time: Time from POEMS onset to stroke onset.

ICA: internal carotid artery. MCA: middle cerebral artery. ACA: anterior cerebral artery. PCA: posterior cerebral artery. BZI: border-zone infarction.

DSA: digital subtraction angiography. MRA: magnetic resonance angiography. CTA: computed tomography angiography.

ASCT: autologous stem cell transplant.

N.A.: not available.

**Table 3:** Comparison of clinical feature in the present study and two previous series

Characteristics	Present study *N* = 28	Li [18] *N* = 99	Dispenzieri [41] *N* = 99
Age (median, years)	47	45	51
Male sex	14 (50%)	58 (58%)	62 (62%)
Initial symptoms			
Polyneuropathy	8 (33%)	41 (41%)	N.A.
Peripheral edema	1 (4%)	27 (27%)	N.A.
Skin changes	4 (19%)	15 (15%)	N.A.
Serositis	3 (14%)	4 (4%)	N.A.
Organomegaly	2 (10%)	3 (3%)	N.A.
Ischemic stroke	4 (19%)	0 (0%)	N.A.
Polyneuropathy	28 (100%)	98 (99%)	99 (100%)
Elevated CSF protein	15 (94%)	69 (96%)	99 (100%)
Organomegaly	26 (93%)	85 (86%)	N.A.
Hepatomegaly	14 (50%)	47 (47%)	24 (24%)
Splenomegaly	21 (75%)	70 (71%)	22 (22%)
Lymphadenopathy	17 (61%)	74 (75%)	26 (26%)
Castleman’s Disease	7 (64%)	25 (58%)	11 (73%)
Endocrinopathy	23 (82%)	N.A.	73 (74%)
Thyroid dysfunction	16 (57%)	66 (66%)	26 (26%)
Impaired glucose metabolism	3 (11%)	21 (21%)	3 (3%)
Monoclonal plasma cell dyscrasia	25 (89%)	99 (100%)	99 (100%)
IgA-λ	10 (40%)	65 (71%)	44 (52%)
IgG-λ	8 (32%)	20 (22%)	40 (48%)
IgM-λ	0 (0%)	0 (0%)	1 (1%)
λ	2 (8%)	4 (4%)	0 (0%)
IgG-κ	1 (4%)	2 (2%)	0 (0%)
IgA-κ	1 (4%)	1 (1%)	0 (0%)
Skin changes	26 (93%)	89 (90%)	67 (68%)
Papilledema	7 (25%)	56 (64%)	29 (29%)
Extravascular volume overload	24 (86%)	87 (88%)	29 (29%)
Peripheral edema	18 (64%)	84 (85%)	24 (24%)
Pleural effusion	12 (43%)	43 (43%)	3 (3%)
Pericardial effusion	12 (43%)	64 (65%)	1 (1%)
Ascites	9 (32%)	54 (55%)	7 (7%)
Sclerotic bone lesions	12 (43%)	19 (19%)	46 (46%)
Other features			
Thrombocytosis	17 (61%)	54 (55%)	53 (54%)
Polycythemia	5 (18%)	9 (9%)	18 (18%)
Hyperfibrinogenemia	6 (21%)	N.A.	N.A.
Pulmonary hypertension	3 (11%)	36 (36%)	5 (5%)
Renal impairment	3 (11%)	37 (37%)	4 (4%)
Elevated VEGF	5 (100%)	28(70%)	N.A.
Surival rate	8^a^ (44%)	86^b^ (83%)	64^c^ (75%)

Values are given as number (percent), unless otherwise indicated. The percentages for subcategories are demonstrated as the percentage of patients with the abnormality among those patients who finished the test .

N.A.: not available.

^a^ longest follow-up time of 2 years ^b^ median follow-up time of 26 months

^c^ median follow-up time of 70.6 months

## GENERAL CHARACTERISTICS

There were 14 males and 14 females, and the male to female ratio was 1:1. The age of ischemic stroke onset ranged from 24 to 68 years (median age=47 years) without a significant difference between male and female (p=0.303). The median time interval between stroke onset and POEMS syndrome onset was six months. The time interval ranged from 12 months preceded the onset of POEMS syndrome to 11 years after the onset of POEMS syndrome. There were no relevant data in two cases. The data of initial symptom was not documented in 4 cases. The most common initial symptom was polyneuropathy (8, 33.3%). Other initial symptoms included ischemic stroke (4, 16.7%), skin changes (4, 16.7%), polyserositis (3, 12.5%), organomegaly (2, 8.3%), impotence (1, 4.2%), diarrhea (1, 4.2%) and headache (1, 4.2%). Most patients (18, 64.3%) had no risk factors of stroke. Meanwhile, twenty-one patients (75.0%) had not any relevant comorbidities of stroke, only a small proportion of patients complained of hypertension (7, 25.0%), smoking (4, 14.3%), diabetes mellitus (3, 10.7%), hyperlipidemia (2, 7.1%), pulmonary embolism (2, 7.1%), myocardial infarction (1, 3.6%), foot gangrene (1, 3.6%), patent foramen ovale (1, 3.6%), Hashimoto’s thyroiditis (1, 3.6%), and previous ischemic stroke (1, 3.6%). Medications before stroke onset which might influence on the risk of ischemic stroke included aspirin (7, 25.0%), glucocorticoids (8, 28.6%) and oral anticoagulants (2, 7.1%).

## POEMS FEATURES

All the patients had slight to severe symptoms and signs of polyneuropathy. In the 16 patients who were reported to receive lumber puncture, 15 patients (93.8%) had elevated cerebrospinal fluid protein levels. Increasing cerebrospinal fluid opening pressure was observed in 4 patients (25.0%). Abnormal white cell count was not observed in any patient. In addition, oligoclonal bands were positive in 3 patients (15.0%).

Twenty-six patients (92.9%) had organomegaly, in which 14 patients (50.0%) had hepatomegaly, 21 (75.0%) had splenomegaly, and 17 patients (60.7%) had lymphadenopathy. Seven patients (25.0%) had both hepatosplenomegaly and lymphadenopathy. Eleven of these patients underwent lymph node biopsy, four patients (36.4%) of them had normal manifestation and 7 patients (63.6%) had Castleman’s disease.

Skin manifestations were observed in 26 patients (92.9%). Hyperpigmentation was the most common manifestation occurring in 23 patients (82.1%), which was followed by skin thickening (8, 28.6%) and hypertrichosis (3, 10.7%). Raynaud’s phenomenon occurred in 3 patients (10.7%). In our study, twenty-three patients (82.1%) had at least one recognized endocrinopathy, with thyroid dysfunction (17, 60.7%) as the most common abnormality. Among the patients with thyroid dysfunction, 14 patients (82.3%) needed replacement treatment with high TSH and low serum thyroxine, 2 patients (11.8%) presented with subclinical hypothyroidism while one patient (5.9%) had hyperthyroidism. Hypogonadism (14, 50.0%) was also a common endocrinopathy in patients of POEMS complicated with ischemic stroke. However, only three patients (10.7%) had impaired glucose metabolism.

The evidence of monoclonal plasma proliferative disorder was found in 25 patients (89.3%). The types of monoclonal protein in these patients consisted of IgA-λ (10, 40.0%), IgG-λ (8, 32.0%), λ-light chain alone (2, 8.0%), IgA-κ (1, 4.0%), and IgG-κ (1, 4.0%). The type of monoclonal protein was not reported in 3 patients. Bone marrow aspiration and biopsy were undertaken in a half of patients (50%). Only one patient had >10% plasma cell.

A variety of patterns of extravascular volume overload was reported in 24 patients (85.7%). Peripheral edema (18, 64.3%) was the most common manifestation in our cases, but pleural effusion (12, 42.9%), pericardial effusion (12, 42.9%) and ascites (9, 32.1%) were also common. In addition, polyserositis was observed in 10 patients (35.7%). Osteosclerotic lesions was documented in 12 patients (42.9%). Papilledema was observed in 7 patients (25%). Eight patients suffered from weight loss and three patients had pulmonary hypertension.

## LABORATORY FEATURES

Thrombocytosis was a very common manifestation in POEMS patients with ischemic stroke. It was found in 17 patients (60.7%). The abnormal platelet count varied from 329×10^9^/L to 1000×10^9^/L. Polycythemia (5, 17.8%) was more common than anemia (2, 7.1%). Hyperfibrinogenemia was documented in 6 patients (18.5%) while elevated CRP levels in 9 patients (33.3%), elevated ESR in 8 patients (28.3%), hypoalbuminemia in 3 patients (10.7%), renal impairment in 3 patients (10.7%), hyperglobulinemia in 3 patients (10.7%), proteinuria in 3 patients (10.7%) and hyperlipidemia in 2 patients (7.1%). In the five patients who performed the measure of the serum VEGF level, all of them had elevated serum VEGF level. In addition, hyperhomocysteinemia was observed in 2 patients (7.1%). Only two patients had autoantibody abnormality, all of them were antinuclear antibody.

## ISCHEMIC STROKE FEATURES

Eleven patients (39.3%) experienced more than one event of ischemic stroke. The highest frequency of ischemic attack was more than 4-5 times per day. In our study cohort, multiple ischemic lesions (20, 71.5%) were much more common than single ischemic lesion (8, 28.5%). Evaluation of cerebral arteries was performed by magnetic resonance angiography (9, 36.0%), digital subtraction angiography (7, 25.0%), ultrasonograph (7, 25.0%) and CT angiography (2, 8.0%) in 25 patients. Nineteen patients (76.0%) were found with cerebral vasculopathy while 12 patients (48.0%) had more than one artery involved. It was noteworthy that mismatch between the infarct area and recognized vasculopathy was observed in 8 patients (28.6%). In addition, extracerebral vasculopathy was found in 9 patients (32.1%).

## TREATMENT AND OUTCOME

Data of treatment was available in 25 patients (88.9%) of all the patients. Among them, five patients underwent POEMS therapy with melphalan-based chemotherapy, five patients with prednisone or dexamethasone alone, three patients with chemotherapy and autologous stem cell transplantation, two patients with lenalidomide and dexamethasone, one patient with thalidomide and dexamethasone, one patient with cyclophosphamide, one patient with prednisone and percutaneous radiotherapy. Eight patients underwent subsequent treatment due to the poor response to the initial treatment. Data of stroke therapy was available in 22 patients. Among them, 13 patients were treated with single antiplatelet drug (11 cases with aspirin, 1 case with a sequential treatments of aspirin and clopidogrel, 1 case without detail in the report), 2 patients with aspirin and clopidogrel, 2 patients with antiplatelet and anticoagulation therapy (2 cases with aspirin and oral anticoagulation, 1 case with clopidogrel and phenprocoumon) , 2 patients with anticoagulation therapy, 1 patient with a sequential treatment of defibrase, ozagrel and aspirin, 1 patient with aspirin and bypass surgery. Ischemic events were documented in 8 patients even when they were undergoing all the therapy for ischemic stroke, but in 3 cases of them, ischemic attack stopped after the combined treatment for ischemic stroke and POEMS syndrome. Survival data was available in 18 patients with a longest follow-up time of 2 years, ten patients died in total. The comparison of the baseline characteristics of patients with and without fatal outcome was showed in Table 4. Due to the rare occurrence of ischemic stroke in patients with POEMS syndrome, the sample size was too small to establish any meaningful difference of clinical features between patients with and without fatal outcome. Patients with fatal outcome were older, and more likely to have multiple cerebral vasculopathies, extracerebral pathology, hepatomegaly, hepatosplenomegaly, papilledema, polyendocrinopathy, and extravascular volume overload. Moreover, very few patients in survived group had extracerebral pathology, hepatomegaly hepatosplenomegaly, papilledema, pericardial effusion, ascites and polyserositis.

**Table 4:** Comparison of clinical features in the patients with and without fatal outcome

Characteristics	Death (*N* = 10)	Survived (*N* = 8)
Age ( median, years)	52	46
Male	6 (60.0%)	5 (62.5%)
Multiple ischemic lesions	8 (80.0%)	6 (75%)
Cerebral vasculopathy	6 (66.7%)	4 (57.1%)
Multiple cerebral vasculopathies	5 (55.6%)	2 (28.6%)
Recurrent stroke	4 (40.0%)	3 (37.5%)
Extracerebral pathology	5 (50.0%)	1 (12.5%)
Polyneuropathy	10 (100%)	8 (100%)
Elevated CSF protein	6 (60.0%)	4 (50.0%)
Organomegaly	10 (100%)	7 (87.5%)
Hepatomegaly	7 (70.0%)	1 (12.5%)
Splenomegaly	8 (80.0%)	5 (62.5%)
Lymphadenopathy	6 (60.0%)	4 (50.0%)
Hepatosplenomegaly	6 (60.0%)	1 (12.5%)
Endocrinopathy	9 (90.0%)	5 (62.5%)
Thyroid dysfunction	6 (60.0%)	4 (50.0%)
Polyendocrinopathy	6 (60.0%)	3 (37.5%)
Skin changes	9 (90.0%)	6 (75.0%)
Papilledema	6 (60.0%)	0 (0%)
Extravascular volume overload	10 (100%)	5 (62.5%)
Peripheral edema	9 (90.0%)	3 (37.5%)
Pleural effusion	6 (60.0%)	2 (25.0%)
Pericardial effusion	6 (60.0%)	1 (12.5%)
Ascites	5 (50.0%)	0 (0%)
Polyserositis	6 (60.0%)	0 (0%)
Sclerotic bone lesions	7 (70.0%)	4 (50.0%)
Thrombocytosis	9 (90.0%)	6 (75.0%)
Hyperfibrinogenemia	1 (10.0%)	0 (0%)

Values are given as number (percent), unless otherwise indicated. The percentages for subcategories are demonstrated as the percentage of patients with the abnormality among those patients who finished the test.

## DISCUSSION

POEMS syndrome is a rare multi-systemic paraneoplastic disease resulting from an underlying clonal plasma cell disorder, which is characterized by the acronym: polyneuropathy, organomegaly, endocrinopathy, mono-clonal plasma cell disorder, and skin changes [2]. It’s also well known to be associated with a myriad of clinical presentations including Castleman’s disease, sclerotic bone lesions, pulmonary manifestations, clubbing, weight loss, thrombocytosis, polycythemia, extravascular volume overload and elevated VEGF levels [1]. The pathophysiologic correlates between the myriad of symptoms and the underlying disease have not yet been fully understood, but the correlation has been postulated that cytokines or proangiogenic factors play a pivotal role as mediators [7-9, 17]. Due to its complicated manifestations and atypical initial presentations of POEMS syndrome, misdiagnosis is very common and extensive [18], which is sometimes a major challenge for physicians. Besides the manifestations commonly seen in this syndrome, arterial or venous thrombosis is attracting increasing attention as a less-recognized feature which is suggested to be a predictor of poor prognosis [8, 10, 11, 14]. So far, several cases of ischemic stroke in patients with POEMS syndrome have been reported in the literature [8, 10, 12-15, 19-39].

Our patient was a 65-year-old female. She presented polyneuropathy, monoclonal protein, splenomegaly, lymphadenopathy, hypothyroidism, extravascular volume overload (peripheral edema, pericardial effusion, pleural effusion and ascites) and skin changes. She also showed Castleman disease, papilledema, elevated serum VEGF and weight loss. According to the diagnosis criteria recommended by Dispenzieri [1], this patient fulfilled two mandatory major criteria, two other major criteria and six minor criteria. The diagnosis was established after excluding other differential diagnoses. In spite of the absence of traditional and modifiable atherosclerosis risk factors such as diabetes, hypertension, hypercholesteremia or smoking, our patient still developed multiple cerebral infarctions under an adequate secondary stroke prevention therapy including aspirin and atorvastatin. Images of CT angiography showed a diffuse stenosis in the large vessels from the origin of left ICA to the M1 segment of the left MCA which is not likely the result of atherosclerosis but a result of POEMS syndrome. The etiological detection for ischemic stroke (complete blood count, coagulation markers, blood chemistry, serum tumor marks, antiphospholipid antibody, autoimmune antibodies and screen for vasculitis) was unremarkable except for anemia, thrombocytosis, hyperhomocysteinemia and hyperfibrinogenemia. It’s noteworthy that thrombocytosis and bone marrow plasmacytosis lead to an increased risk of ischemic stroke in Dupont’s study [12]. Thrombocytosis is a well recognized etiologic factor of arterial thrombosis with proposed mechanisms including increased blood viscosity and pre-thromboticstate [10, 40]. However, in our study, there are 11 patients (39.3%) with normal platelet counts. Meanwhile, the incidence of thrombocytosis in our cohort consist of POEMS patients with ischemic stroke is very similar to the two large series studies of POEMS performed by Dispenzieri [41] and Li [18] respectively. Most patients in our study just presented mild or moderate thrombocytosis. Only one patient had a platelet count more than 1000×10^9^/L. In contrast, most studies agreed that platelet count apparently did not correlate with the risk of thrombosis in patients with essential thrombocythemia except the patients with the basis of age above 60 years, cardiovascular risk factors, thrombosis history and the presence of JAK-2 mutation [42, 43]. Even a very high platelet count is associated with a high risk of bleeding but not thrombosis [43]. Also, it is very rare that myeloproliferative and plasma cell proliferation develop in the same patient. It’s postulated that abnormal cytokine hyperproduction such as interleukin-6 (IL-6) and interleukin-1 (IL-1) may be responsible for the muti-systematic manifestations including thrombocytosis and Castleman’s disease [44]. In addition, the recurrence of ischemic stroke was observed in 8 patients even under sufficient oral antiplatelet therapy, which indicated a complex association between POEMS syndrome and ischemic stroke.

Recently, fibrinogen is emerging as an independent risk factor for cardiovascular diseases and peripheral vascular diseases. However, still few studies are available to regard the role of fibrinogen in the pathogenesis of ischemic stroke. Previous studies have shown conflicting results as to whether fibrinogen is a predictor for risk of ischemic stroke [45, 46]. Kang and colleagues found all of their three cases had elevated fibrinogen levels [21]. The phenomenon was also observed in a patient with a large area of cerebral infarction and Castleman’s disease reported by Huang et al [23]. They suggested IL-6 inducing hyperfibrinogenemia might act a pivotal part as a trigger of procoagulatory in the development of stroke in patients with POEMS syndrome [15, 21, 23, 34]. Fibrinogen is a predictor of wide-spread inflammation process and also frequently elevated before the cerebrovascular events in the patients without a history of POEMS, because cytokines such as IL-1, IL-6 and tumor necrosis factor which were demonstrated to be elevated in patients with POEMS syndrome, can also be produced by atherosclerosis macrophages [47, 48, 49]. Moreover, hyperfibrinogenemia is not observed in all patients with elevated cytokine levels. In addition, there were only six patients (21.2%) had elevated fibrinogen levels in our review. From the standpoint of available evidence, the hypothesis of the pivotal role of fibrinogen in the etiologic link between the two disorders is less plausible.

On the other hand, hyperhomocysteinemia was observed in our case and two patients (7.1%) in our review, which may be a critical but often-neglected component of POEMS. Numerous studies have demonstrated that hyperhomocysteinemia is a strong and independent risk factor for ischemic stroke [49, 50]. It is now well recognized that vitamin B12 supplement therapy can reduce homocysteine levels, which will lead to a reduced risk of ischemic stroke [50]. Recently, vitamin B12 deficiency unresponsive to replacement therapy has been recognized as a less common manifestation of POEMS [3, 4]. Although our patient received a sufficient treatment of oral mecobalamin 500ug three times a day, we still detected a low vitamin B12 value and hyperhomocysteinemia without the presence of intrinsic factor antibodies. The pathophysiological mechanisms under vitamin B12 deficiency and hyperhomocysteinemia in POEMS syndrome worth exploring. This phenomenon may be caused by the abnormal uptake in gastric mucosa which may suffer from microangiopathy and vasopermeability induced by VEGF.

Vessel endothelial cell is an important target of cytokines and M protein [8, 10, 17, 51, 52]. It’s noteworthy that large artery occlusive disease in our study is much more common than that reported in stroke registry studies [53, 54, 55]. However, the frequencies of stroke risk factors such as diabetes mellitus, hypertension and atrial fibrillation occurred in our study are similar to or even lower than those in stroke registries [54, 55]. In accordance with previous literature, the preponderance of multiple cerebral vessel lesions and coexistence of extracerebral vessel disease in patients of POEMS without stroke risk factors are also more common than those reported in stroke registries [13-14, 22-24, 27-28, 38]. This phenomenon may suggest that POEMS syndrome has a predisposition towards large-vessel vasculopathy which could only be confirmed by biopsy. To the best of our knowledge, pathologically confirmed cerebral vasculopathy has not been reported yet. Conventional angiographic findings of cerebral vasculopathy are nonspecific and can’t reliably distinguish vasculopathy from atherosclerosis [56]. Magnetic resonance vessel-wall imaging is the most promising technique for differentiating between vasculopathy and atherosclerotic disease [56]. Our patient showed a thickening and enhancement of the vessel wall of the left ICA, indicative of vasculitis. In the previous studies, a noninflammatory vasculopathy, which is speculated to be the pathogeny of stroke according to the benign cerebrospinal fluid manifestation in patients [12, 28], might be due to proangiogenic factors including VEGF. However, the hypothesis is challenged in the absence of pathological evidence [12, 28]. There is growing evidence that endothelial dysfunction plays an important role in the pathogenesis of POEMS syndrome. Moreover, pathologically confirmed vasculitis involving small-sized and medium-sized muscular artery are reported to be directly associated with POEMS syndrome [57, 58]. In addition, larger-sized vessels are also suspected to be affected in POEMS syndrome [8, 10, 22, 59]. Co-existing quasi-moyamoya disease and POEMS syndrome were observed in a patient with sudden-onset intracranial hemorrhage [60]. Progressive intracranial large-vessel vasculopathy has been reported as the initial presenting manifestation of POEMS [28].

The precise pathophysiological mechanism of the development of cerebral vasculopathy remains unknown. An correlation with cerebral vasculopathy and Castleman’s disease in patients with POEMS syndrome was reported in several studies. Castleman’s disease is one of the major criteria for the diagnosis of POEMS syndrome [1]. An association with Castleman’s disease was reported in nearly half of patients with POEMS syndrome. However, the pathogenesis of Castleman’s disease remains unclear. A complex cytokine and chemokine network, including VEGF, IL-6, and IL-1, may play an important role in the development of Castleman’s disease [61]. Also, the network may induce a systemic inflammatory condition which may lead to cerebral vasculopathy and ischemic stroke [62]. Ischemic stroke is the most common manifestation of cerebrovascular involvements of Castleman’s disease. Seven patients were found with Castleman’s disease and ischemic stroke in our study. In the five patients who had undergone an evaluation of cerebral vessels, all of them were found with cerebral vascopathy. It indicated that there was a strong association between cerebral vasculopathy and Castleman’s disease. Hori et al. [38] described a patient with Castleman’s disease and POEMS syndrome who presented hypertrophic cranial pachymeningitis and bilateral internal carotid artery occlusion due to suspected vasculitis. Rössler and colleague [22] reported a patient with Castleman’s disease and POEMS syndrome who presented recurrent ischemic stroke secondary to clinical diagnosed cerebral vasculitis. Garcia et al. [13] described two patients with diffuse stenosis of the intracranial arteries, being suggestive of a non-atherosclerotic cerebral vasculopathy. They hypothesized that cerebral vascopathy might be caused by the overproduction of cytokines, such as VEGF, IL-6, and IL-1. Elevating serum levels of IL-6 is very common in patients with Castleman’s disease [61], and it’s well known as a key factor of the systemic inflammatory and a predictor of poor outcome of ischemic stroke [62]. IL-6 may play a pivotal role in the etiologic link between Castleman’s disease and the occurrence of cerebral vascopathy and ischemic stroke.

Recently, the overproduction of VEGF has been proved to contribute to most of the clinical manifestations and correlate with the disease activity and response to therapy [5, 6]. Meanwhile, VEGF is also suggested to be the key factor of vessel walls damage in POEMS syndrome. VEGF is a potent multifunctional cytokine that potently induces angiogenesis, microvascular hyperpermeability and inflammation on vascular endothelial cells [63, 64]. Elevated serum or plasma VEGF, as one of the major diagnostic criteria of POEMS syndrome, induces plasma exudation and the migration of inflammatory cells into vascular inflammatory lesions via upregulating the expression of procoagulative properties, endothelial adhesion molecules and leukocyte chemokine, which in turns initiate and aggravate endothelial damage [65]. Angiogenesis within the adventitia of vessel walls induced by VEGF, which also plays an important role in the pathogenic of chronic inflammation of vessel walls [7, 8, 64]. The histopathological findings of high VEGF expression on arterial smooth muscle cells may result from the high level of VEGF. On the other hand, the elevated VEGF may be produced not only by VEGF secreted from plasma cells and platelets but also by VEGF exudation from inflamed organs including vessels [63]. In this context, VEGF is suggested to be an important mediator in the development of multiple vasculopathy lesions, and this hypothesis is strengthened by the phenomenon that increased VEGF has been observed in many vasculitic disorders include Takayasu’s arteritis and Behcet’s disease [66].

POEMS syndrome is considered as a chronic disease with a good long-term outcome. Recent data demonstrated that 5-year and 10-years survival rate was 84% and 77%, respectively [67]. It’s documented that only age more than 50 years, pulmonary hypertension, pleural effusion and an eGFR less than 30 ml/min/1.73 m^2^ were independently associated with overall survival [67]. In contrast, we observed fatal outcome in POEMS patients complicated with ischemic stroke. Ten of the eighteen patients who had follow-up data died within two years after stroke events. Even most of them had already received sufficient therapies for ischemic stroke and POEMS syndrome [10, 14, 15, 24, 26, 28]. It suggested that ischemic stroke was a poor outcome predictor in patients with POEMS syndrome. But until now, there is a lack of evidence and standard of the appropriate treatment for the patients who suffer from POEMS syndrome and ischemic stroke simultaneously. The recommended treatments for POEMS syndrome include autologous stem cell transplantation, radiotherapy and melphalan-based chemotherapy [1]. It is worth noting that recurrent ischemic stroke events were interrupted after chemotherapy or corticosteroids therapy in several patients [12, 25, 27]. Immunomodulatory drugs including thalidomide and lenalidomide represent a new treatment option for the patients with POEMS syndrome, not only because of their excellent suppression of monoclonal plasma cell proliferation, but also because of the modulation of VEGF secretion and upregulated cytokines suppressed by these drugs [1, 5, 6, 68]. Meanwhile, VEGF inhibition is currently studied in cardiovascular disease [69] and has also shown neuroprotective effects on cerebral ischemia and reperfusion injury [70]. They have been considered for cerebrovascular disease. Single antiplatelet therapy including aspirin and clopidogrel, as a recommendation of AHA/ASA guideline for stroke secondary prevention [54], showed no effect on stopping ischemic recurrence in many reports [13, 15, 20, 21, 24, 29]. Meanwhile, recurrent events were observed under heparinization or oral anticoagulation [15, 20, 22]. So more aggressive therapy is required. Dual antiplatelet therapy with clopidogrel and aspirin may be a potential treatment, which was reported in some studies [24, 29]. Even though there is an increased incidence of hemorrhage using combination therapy with clopidogrel and aspirin, the risk of ischemic events is markedly reduced in predisposed patients with no response to initial antithrombotic therapy treatment or with established atherothrombotic disease. Despite these advances, the management of POEMS syndrome remains a major challenge, especially in the patients complicated with ischemic stroke.

## LIMITATIONS

Ischemic stroke is a less common manifestation of a rare disease-POEMS syndrome. As a result of the lack of understanding of the association between ischemic stroke and POEMS syndrome, many cases have been misdiagnosed. There have been just a few reports on this disease and most of them are case reports. Also, details were not provided in some of the cases, including vascular imaging data, recurrent event data and survival data. The extremely rare occurrence of ischemic stroke in patients with POEMS syndrome and a comprehensive analysis of literature primarily based on cases reports present challenges in establishing any firm conclusions on pathogenesis, management and prognosis. Further research focusing on larger cohort may help in better characterizing and treating this rare complication of POEMS syndrome.

## CONCLUSION

To the best of our knowledge, the present case of POEMS syndrome is the first to describe to be associated with cerebral vasculitis confirmed by magnetic resonance vessel-wall imaging technique. The underlying cerebral vasculopathy, leading to the occurrence of ischemic stroke, may be induced by the overproduction of cytokines and systemic inflammation due to POEMS syndrome. Magnetic resonance vessel-wall imaging technique, which is the most promising noninvasive technique for differentiating cerebral vasculopathy from atherosclerotic disease, may contribute to an early diagnosis and treatment for patients with POEMS syndrome. Our literature analysis has revealed that ischemic stroke is a poor outcome predictor in patients with POEMS syndrome. A preponderance of multiple cerebral vessel lesions and low survival rate were observed. VEGF may play a critical role in the pathogenesis of POEMS syndrome. It’s also an important mediator in the development of cerebral vasculopathy. Carefully studying ischemic stroke in patients with POEMS syndrome might help to improve the survival rate and the prognosis.
